# Large Deletions at the SHOX Locus in the Pseudoautosomal Region Are Associated with Skeletal Atavism in Shetland Ponies

**DOI:** 10.1534/g3.116.029645

**Published:** 2016-05-19

**Authors:** Nima Rafati, Lisa S. Andersson, Sofia Mikko, Chungang Feng, Terje Raudsepp, Jessica Pettersson, Jan Janecka, Ove Wattle, Adam Ameur, Gunilla Thyreen, John Eberth, John Huddleston, Maika Malig, Ernest Bailey, Evan E. Eichler, Göran Dalin, Bhanu Chowdary, Leif Andersson, Gabriella Lindgren, Carl-Johan Rubin

**Affiliations:** * Department of Medical Biochemistry and Microbiology, Science for Life Laboratory, Uppsala University, Sweden 751 23; † Department of Animal Breeding and Genetics, Swedish University of Agricultural Sciences, Uppsala, Sweden 750 07; ‡ Department of Veterinary Integrative Biosciences, Texas A&M University, College Station, Texas 77845; § Department of Clinical Sciences, Swedish University of Agricultural Sciences, Uppsala, Sweden 750 07; ** Department of Immunology, Genetics and Pathology, Science for Life Laboratory, Uppsala University, Sweden 751 23; †† Gluck Equine Research Center, Department of Veterinary Science, University of Kentucky, Lexington, Kentucky 40546; ‡‡ Department of Genome Sciences, University of Washington School of Medicine, Seattle, Washington 98105; §§ Howard Hughes Medical Institute, University of Washington, Seattle, Washington 98195; *** Department of Anatomy, Physiology and Biochemistry, Swedish University of Agricultural Sciences, Uppsala, Sweden 750 07; ††† New Research Complex, Qatar University, Doha, Qatar 2713

**Keywords:** SMRT sequencing, skeletal atavism, SHOX, PAR

## Abstract

Skeletal atavism in Shetland ponies is a heritable disorder characterized by abnormal growth of the ulna and fibula that extend the carpal and tarsal joints, respectively. This causes abnormal skeletal structure and impaired movements, and affected foals are usually killed. In order to identify the causal mutation we subjected six confirmed Swedish cases and a DNA pool consisting of 21 control individuals to whole genome resequencing. We screened for polymorphisms where the cases and the control pool were fixed for opposite alleles and observed this signature for only 25 SNPs, most of which were scattered on genome assembly unassigned scaffolds. Read depth analysis at these loci revealed homozygosity or compound heterozygosity for two partially overlapping large deletions in the pseudoautosomal region (PAR) of chromosome X/Y in cases but not in the control pool. One of these deletions removes the entire coding region of the SHOX gene and both deletions remove parts of the CRLF2 gene located downstream of SHOX. The horse reference assembly of the PAR is highly fragmented, and in order to characterize this region we sequenced bacterial artificial chromosome (BAC) clones by single-molecule real-time (SMRT) sequencing technology. This considerably improved the assembly and enabled size estimations of the two deletions to 160−180 kb and 60−80 kb, respectively. Complete association between the presence of these deletions and disease status was verified in eight other affected horses. The result of the present study is consistent with previous studies in humans showing crucial importance of SHOX for normal skeletal development.

Hereditary skeletal anomalies have been described in certain breeds of horse, including lateral patellar (sub)luxation in Shetland ponies (Hermans *et al.* 1987) and dwarfism with disproportional back and short limbs in Friesian horses (Back *et al.* 2008; Orr *et al.* 2010), but in neither of these cases has a causal variant been reported. It has however been suggested that mutations in Aggrecan (ACAN) are associated with chondrodysplasia-like dwarfism in miniature horses (Eberth *et al.* 2009).

Shetland ponies born with abnormally developed ulna and fibula have been described since the 1950s (reviewed in Hermans 1970). In horses, “full length” development of fibula and ulna to include the carpal and tarsal joint, respectively, results in splayed legs and movement difficulties (Figure 1). Shorter than normal humerus, femur, and tibia, in relation to the third metatarsal bone, are also observed and affected individuals usually have to be killed at an early age (Tyson *et al.* 2004). Fossil records show that approximately 15 million yr ago, in the ancestors of modern equids, ulna and fibula were reduced in size and were fused to the radius and tibia, respectively (reviewed in Hall 1995; Tyson *et al.* 2004). The reappearance of properties previously seen at an earlier evolutionary stage of a species is referred to as an atavism (Hall 1995) and the disease in Shetland ponies has therefore been referred to as skeletal atavism (SA). Other examples of atavisms include hind limbs in whales (Tomic and Meyer-Rochow 2011), hypertrichosis (excess of hair) in human (DeStefano *et al.* 2013), and polydactyly in horses (Carstanjen *et al.* 2007).

**Figure 1:**
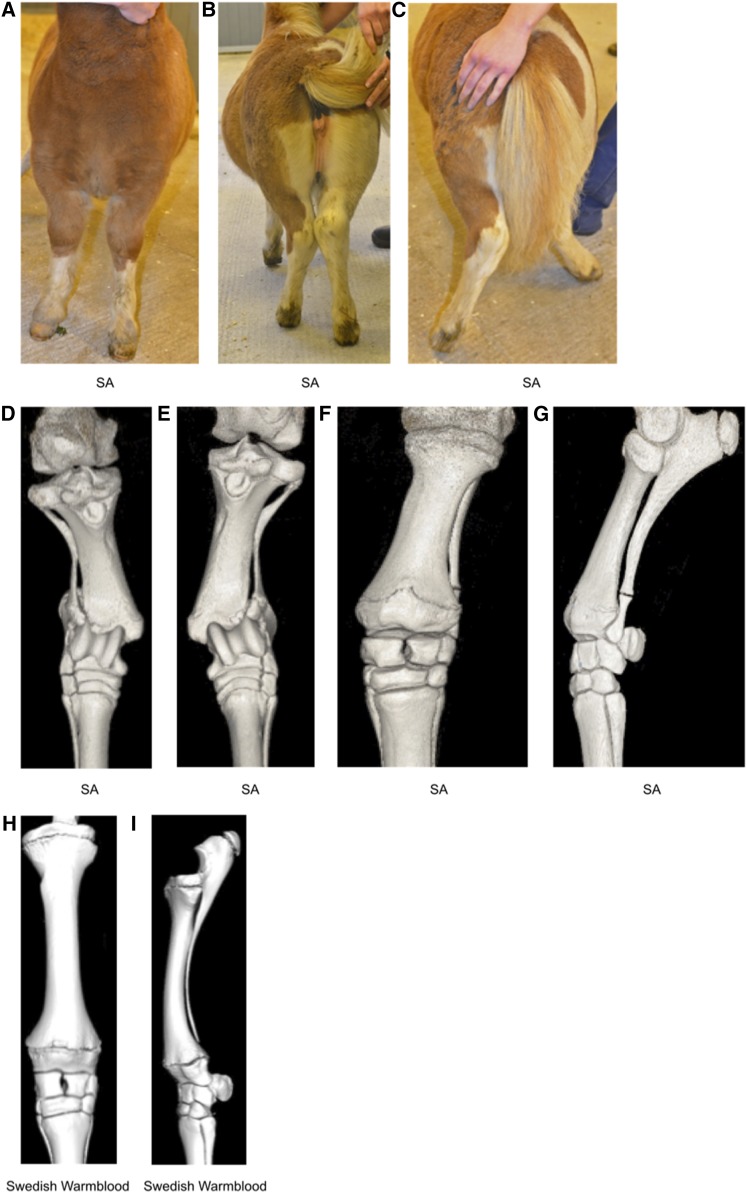
Limbs of a 16-wk-old Shetland pony with skeletal atavism. (A) View from the front when standing square, (B) caudal view when standing, and (C) caudal view at walk. Complete fibulas and ulnas cause instability in the tarsocrural and antebrachiocarpal joints, respectively; angular limb deformities become more severe at walk. (D–G) Computed tomography scans of the 16-wk-old Shetland pony’s gaskin and forearm. Dorsal views of tibia and complete fibula, right (D) and left (E) hind limbs. (F) Dorsal and (G) lateral views of left front limb radius and complete ulna. (H) Computed tomography scans showing dorsal and (I) lateral views of normally developed radius and ulna, with the ulna about to be fused to the radius, of a 16-wk-old nonatavistic Swedish Warmblood foal.

The first affected Shetland ponies were reported in 1958 in the UK where lameness and limb deformities were observed in some pedigrees (Tyson *et al.* 2004), and previously published data have been consistent with an autosomal recessive inheritance (Hermans 1970; Hermans *et al.* 1987; Tyson *et al.* 2004). Due to SA occurring in the UK, Netherlands, and Sweden since the 1960s, it is important to reveal the genetic basis of the disease and to develop a diagnostic test that can be used to avoid mating disease allele carriers. In a first attempt to unravel the genetic basis of this disorder we performed a genome-wide association study (GWAS) of 72 individuals (36 controls, 22 carriers, and 14 cases) using the Illumina EquineSNP50 BeadChip. This analysis did not reveal any association between genetic markers and disease status. Instead, we performed whole genome resequencing of six SA cases and a pool of control horses and use this data to show that skeletal atavism is associated with two, partially overlapping, large deletions on sequence scaffolds not assigned to any chromosome in the EquCab2.0 genome assembly. The genotyping array used for the GWAS had no markers near this associated region. One of the identified deletions removes the entire coding region of the *short stature homeobox* (*SHOX*) gene and both deletions remove parts of the *cytokine receptor-like factor 2* (*CRLF2*) gene located downstream of *SHOX*.

## Materials and Methods

### Animals

The study was approved by the Ethics Committee for Animal Experiments in Uppsala, Sweden (application number C121/14).

### Swedish samples

#### Samples subjected to whole genome sequencing:

Individuals selected for control pool inclusion (*n* = 21) were stallions having no confirmed atavistic offspring despite having sired at least 50 registered offspring. Six SA cases, two of which had been confirmed by X-ray, were sequenced individually.

#### Samples selected for validation:

Obligate carriers (*n* = 17) were selected based on confirmed SA affected offspring (of which four did not have an offspring among tested cases), while potential carriers (*n* = 15) were selected because they were close relatives of known or unverified carriers (*n* = 10) or parents of unverified cases (*n* = 5). In addition to the SA cases subjected to whole genome resequencing, we genotyped four other Swedish cases, three of which had been confirmed by X-ray. The random set consisted of 94 Shetland ponies randomly selected from the biobank at the Animal Genetics Laboratory, SLU, Sweden. The horses were born between 1968 and 2000 (average birth year 1991, 70% of the individuals were born in the 90s) and consisted of 72 males and 22 females. For the height study we also used all individuals with available height and sex data (except cases). In addition, we could infer the genotype of seven horses and genotyped eight extra horses collected from the biobank because both DNA and height data were available.

#### American samples:

In total, 18 US horses were genotyped. Four cases were identified by horse owners and samples submitted in connection with investigations of another form of congentital dwarfism (Eberth *et al.* 2009). Photographs were used to identify atavistic samples, and to confirm phenotypic characteristics. Only one carrier, confirmed by producing atavistic offspring, was included. Controls were individuals with no confirmed cases of producing atavistic offspring.

### BAC clone selection, sequencing, and assembly

BAC libraries were generated from a half-brother (“Bravo”) of the female Thoroughbred horse (“Twilight”) used for the EquCab2.0 genome assembly. This BAC library (CHORI-241) was developed by BACPAC Resource center (BPRC) at Children’s Hospital Oakland Research Institute (CHORI), Oakland, CA. We first aligned the BAC end sequences to the deleted regions and selected clones overlapping parts of the deleted regions. In total we sequenced nine BAC clones: 194E12, 288L23, 50P17, 52P20, 159K1, 442L16, 291B18, 712C2, and 419P11 (Supplemental Material, Table S3).

After assembly, for quality control, we checked the coverage of assembled contigs and screened for contaminations by aligning the contigs on UniVec (Version 2012-09-24). After aligning the assemblies against EquCab2.0, we generated consensus sequences based on overlapping similarity between BAC clone assemblies and coordinates on EquCab2.0. We compiled three contigs based on assemblies of five out of the nine BACs covering regions surrounding *SHOX* and used these contigs for downstream analysis (Table S4 and Figure 4A).

We aligned three BAC-derived consensus contigs to human chrX (GRCh37) by MUMmer 3.0 (Kurtz *et al.* 2004) and then identified regions showing sequence conservation.

### SMRT BAC clone sequencing and assembly (Washington School of Medicine)

DNA was isolated from CHORI-241 BAC clones. PacBio SMRTbell libraries were prepared (Travers *et al.* 2010) and sequenced on a RSII with P4-C2 chemistry (one SMRT cell/BAC sample with one 120 min movie). Inserts were assembled using hierarchical genome-assembly process (HGAP) and Quiver as previously described (Chin *et al.* 2013; Huddleston *et al.* 2014).

### SMRT BAC clone sequencing and assembly (SciLife Lab, Uppsala)

SMRTbell libraries were produced using the Pacific Biosciences 1.0 template preparation kit according to the manufacturer’s instructions. SMRTbell were constructed and sequenced following the recommended Pacific Biosciences 2-kb template preparation protocol. In brief, the BACs (500−700 ng) were sheared into 2-kb fragments by the Covaris S2 system using clear miniTUBEs following the manufacturer’s recommendations. The sheared DNA then underwent end-repair and adaptor ligation processes to generate SMRTbell libraries for circular consensus sequencing. Libraries were then subjected to exonuclease treatment and Ampure bead wash procedures for cleanup. SMRTbell libraries were quantified using the Qubit assay and library size was confirmed using the Bioanalyzer 12000 kit. Following SMRTbell construction, v2 primers and P4 polymerase were annealed and the enzyme bound complexes attached to magnetic beads for loading. Each SMRTbell library was loaded on to one SMRT cell and sequenced on the PacBio RSII instrument using C2 chemistry and a 240 min movie time. The resulting reads were *de novo* assembled into contigs using the HGAP algorithm (Chin *et al.* 2013) available in the SMRT analysis portal.

### Resequencing, alignment, and SNP calling

Using standard protocols, we isolated DNA from blood of six affected horses and 21 male Swedish Shetland ponies with no history of having sired affected foals, and combined equimolar quantities of DNA from these in a DNA pool. One paired-end Illumina sequencing library was generated for each case and two libraries were constructed for the control pool by applying a standard protocol at the SNP&Seq Illumina platform at the Science for Life laboratory at Uppsala University, Sweden. These libraries were sequenced as paired-end reads (2 × 100 bp) by Illumina Hiseq2000 (Table S1). We mapped reads of all samples on the horse draft genome assembly (EquCab2.0) using the Burrows-Wheeler alignment (BWA) software (Li and Durbin 2010). After marking duplicated reads by Picard (v1.92 http://picard.sourceforge.net) we realigned reads around InDels and called SNPs by GATK UnifiedGenotyper (McKenna *et al.* 2010). In order to screen for sequencing depth of coverage we first extracted per-position genome-wide depths of each sample by the GATK command DepthOfCoverage and then calculated normalized depths for 1-kb windows along the genome using custom python scripts available at github. We used allele-specific read counts at SNPs and depth of coverage data for our first screen where we identified regions where cases consistently deviated from the control pool with regard to allele frequencies at SNPs or normalized depth.

In order to identify new variation based on new BAC clone assemblies we first combined BAC-derived consensus sequences with EquCab2.0 excluding overlapping sequences with more than 50% similarities to BAC-derived consensus sequences. We followed the same pipeline as for alignments on EquCab2.0 but we recalibrated our alignments with available horse SNP from Ensembl77 before calling SNPs and InDels. After filtering the data based on GATK best practice filters we identified 9,844,628 SNPs and 1,111,009 InDels.

### GenScan on SMRT sequencing contigs

We scanned the BAC-derived consensus contigs for possible protein coding genes using GenScan (Burge and Karlin 1997). Then we aligned the predicted peptide sequences to the nr database by BLASTp (Altschul *et al.* 1990) and visualized the corresponding regions on human chrX. One of the identified protein coding genes was *CRLF2*, which aligned on human chrX and part of this prediction was located inside of Del-2. In order to characterize the coding sequence more precisely we used data from 24 RNA-seq runs (SRA number: SRP012972) generated from blood and muscle of six Thoroughbred horses before and after exercise (Kim *et al.* 2013). After trimming the reads by trimmomatic (Bolger *et al.* 2014), we aligned the RNA-seq reads to the alternative EquCab2.0 genome by GSNAP (Wu and Nacu 2010) and then assembled the transcripts by cufflinks (version 2.2.1) (Trapnell *et al.* 2010). The assembled transcripts on BAC-C3 were translated to protein sequence by Emboss Transeq (Goujon *et al.* 2010) and resulting protein sequences were then aligned to UniProt database (UniProtConsortium 2015) (downloaded on 18 October 2015) by BLASTp.

### Genotyping

We genotyped a group of Swedish and American samples including cases, obligate/potential carriers, and controls by using TaqMan assays designed based on Del-1 and Del-2 sequences from EquCab2.0. As reference assay we used RNAseP (Table S6). Reactions contained 20 ng DNA that was not digested with any restriction enzyme but otherwise analyzed as described below, using the ddPCR platform.

We also genotyped a random set of Shetland ponies (94 individuals) and individuals with known phenotypes by using TaqMan assays designed from BAC-derived consensus contigs on a droplet ddPCR instrument. In this experiment we used an assay targeting *myostatin* (*MSTN*) as a reference, three assays targeting Del-1 sequences, and three assays targeting Del-2 sequences (Table S6). The sequences of Del-1 and Del-2 are highly polymorphic, which may affect assay performance. Thus, we consulted Illumina read alignments to place primers and probes in nonpolymorphic regions and also designed three assays for each deletion to use alternative assays in case genotyping results appeared unreliable.

The ddPCR experiments were performed using the Bio-Rad QX100 ddPCR platform. We first digested the DNA with FastDigest Eco47I restriction enzyme (Thermo Scientific). The final reaction contained 33 ng digested DNA, 900 nM of each primer, and 250 nM of each probe. Twenty microliters of the reaction was loaded into a droplet generator cartridge. Droplets were generated following the manufacturer’s suggested protocol. Cycling conditions were 95° for 10 min, followed by 40 cycles of 94° for 30 sec and 60° for 1 min, and a final 10 min at 98°. The PCR plate was transferred to QX100 droplet reader for reading and the data were analyzed using the software QuantaSoft. For ambiguous events, we used the ellipse, rectangle, and Lasso threshold in order to adjust the classification of clusters. To classify genotypes of individuals we considered a range for the measured copy numbers as follows: two copies (1.7–2.3), one copy (0.7–1.3), and for null copy we did not find any outliers to adjust the range.

We performed ANOVAs to test the association between height, deletion genotypes and sex by using the following models:

Height=genotype_class+sex (1)

where genotype_class consists of Del_carriers (Del-1/wild type (WT) and Del-2/WT together) and WT/WT.

Height=genotype+sex (2)

where genotype consists of Del-1/WT, Del-2/WT, and WT/WT.

### Data availability

All the sequencing data are available from National Center for Biotechnology Information (NCBI) under the accession number PRJNA303134. The python script used to calculate normalized depth of coverage is available for download at https://github.com/cjrubinlab/python_scripts.git (CalcCovGATKDepthFiles_1kb.py).

## Results

### Genome resequencing reveals complete association between disease status and the presence of two deletions in the pseudoautosomal region (PAR)

**Figure 2:**
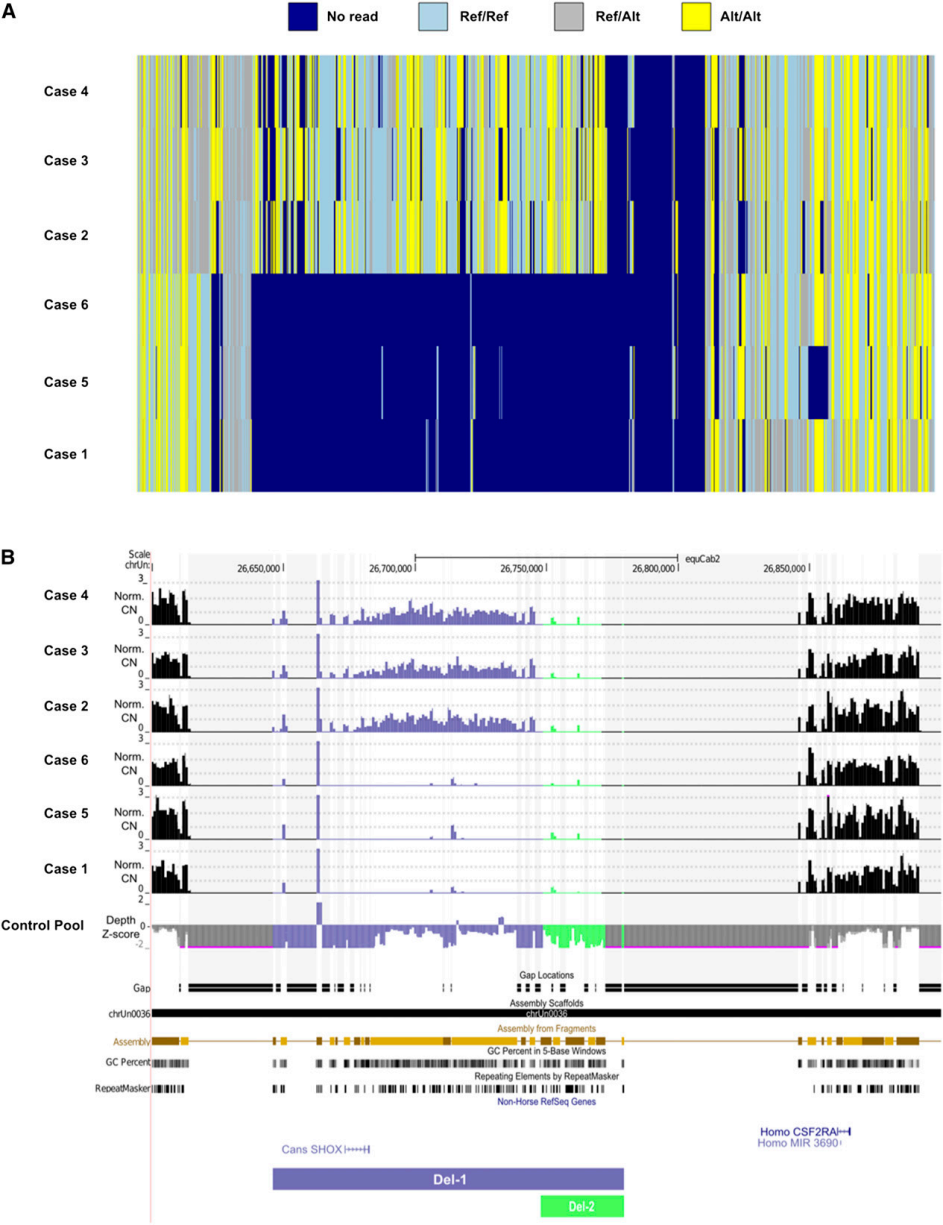
SNP genotypes and depth pattern on EquCab2.0 unassigned scaffold chrUn0036. (A) SNP genotypes of SA cases. (B) Normalized copy numbers of SA cases in relation to the control pool and Z-score transformation of control pool depth in relation to the average depth over the entire genome. The region shown is chrUn: 26.6−26.9 Mb in UCSC genome browser concatenation of unassigned scaffolds.

Our first screen for causal alleles involved GWAS using the EquineSNP50 BeadChip, which did not reveal any association between genetic markers and disease status. As an alternative we selected six affected individuals and a pool of control individuals for whole genome resequencing. For the control pool, we selected males without known history of siring affected foals. We generated 7X sequence coverage for each case and 56X coverage for the control pool (Table S1). More than 94% of the reads were aligned to the equine reference genome and we scanned these alignments to identify SNPs and small Insertion/Deletions (InDels). Since the mode of inheritance of the disease was expected to be autosomal recessive we identified all SNPs with an allele frequency difference of 1.0 between cases and controls and where the control pool only had reference alleles. Two SNPs on anchored chromosomes (chr1) and 23 SNPs on unassigned scaffolds fulfilled the sought pattern, but only for cases 2, 3, and 4, with the other three samples having no reads spanning these SNP positions. Seventeen out of the 23 SNPs on unassigned scaffolds were located on contigs belonging to assembly scaffold chrUn0036, and spanned between chrUn:26,645,953-26,779,752 (EquCab2.0 UCSC genome browser coordinates), a region containing the *SHOX* gene. Careful examination of genetic variants and sequence read depth in these contigs revealed a consistent difference for several unassigned scaffolds where three of the SA cases (2, 3, and 4) were homozygous for variant alleles while the other three SA cases were entirely lacking sequence reads. In contrast, the control pool had normal depth of coverage (Figure 2, A and B). We further concluded from read depth analyses that three SA cases (1, 5, and 6) were homozygous for a large deletion over this region. The other three SA cases (2–4) showed approximately 50% reduction in read depth over most of this deletion but were also deleted for a segment overlapping the first deletion. These two deletions were scattered on chrUn (scaffolds that were not assigned to chromosomes in the EquCab2.0 genome assembly) removing ∼160 kb of EquCab2.0 sequence in cases (Table S2). We named the larger deletion Del-1 and the smaller Del-2. We observed a consistent depth difference on unassigned scaffold chrUn0036, containing 19 contigs (EquCab2.0 UCSC genome browser coordinates chrUn: 26,650,752–26,745,752) (Figure 2B). The heterogeneity of genotypes of cases 2–4 in this region in Figure 2A is due to less stringent filters applied to call SNPs in hemizygous cases.

**Figure 3:**
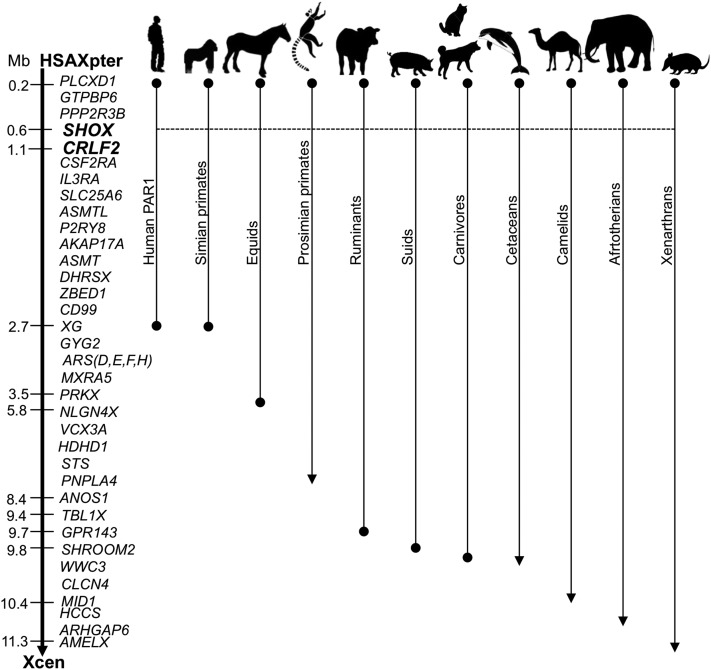
The organization of the PAR in eutherian species. The gene map of HSAXp (left) serves as a reference (see Raudsepp and Chowdhary 2016).

The chrUn0036 scaffold did not contain any complete equine Ensembl protein annotation or RefSeq annotation, but we observed reliable alignments to *SHOX* RefSeq gene models from other species as visualized by *Canis familiaris SHOX* alignment. SHOX is a homeobox transcription factor involved in growth and expressed during development (Benito-Sanz *et al.* 2012). *SHOX* is located in the PAR of most eutherian mammals (Raudsepp *et al.* 2012) (Figure 3) but is absent from rodent genomes (Benito-Sanz *et al.* 2012). We concluded that Del-1 deletes the entire *SHOX* gene and that Del-2 is located proximal of *SHOX* in a region possibly containing *SHOX* regulatory elements.

### A long-read assembly of the SHOX regions

**Figure 4:**
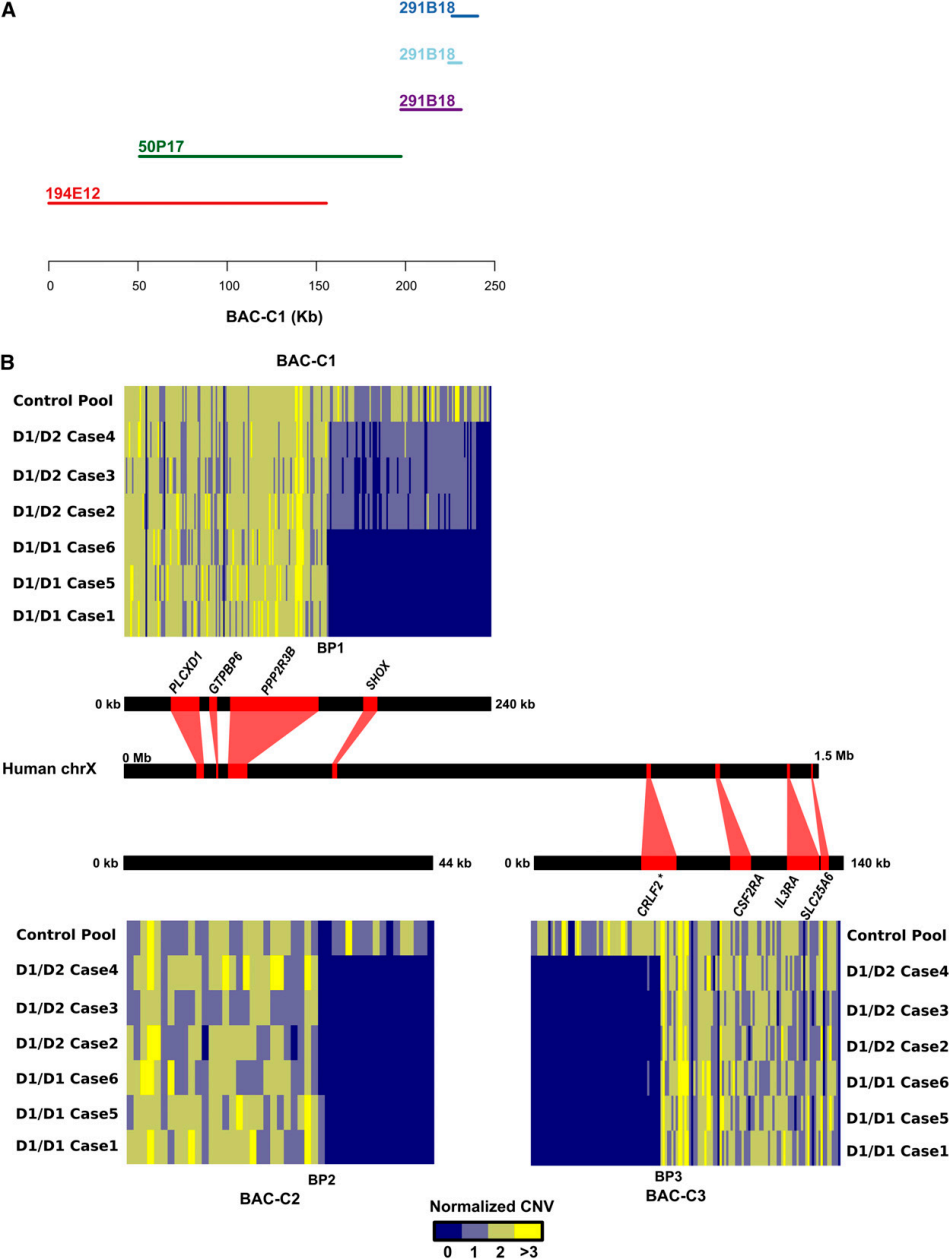
Depth of coverage for cases and the control pool along SMRT sequencing-derived consensus sequences. (A) BAC assembly contigs used to generate consensus sequence BAC-C1. BAC 291B18 assembled into three contigs. (B) Normalized copy numbers observed in 1-kb windows along BAC-contigs BAC-C2 and BAC-C3 as well as BAC-derived consensus contig BAC-C1. Syntenic similarity to human is visualized as red lines combining each contig/BAC-derived consensus sequence with coordinates on the PAR of human ChrX. BAC-C2 did not have any syntenic similarity to human Chr. X. BP1-3 indicate predicted deletion breakpoints; long stretches of TGGA repeats occurred at both BP1 and BP3. Predicted protein coding genes with similarity to human genes are visualized using red boxes. *The *CRLF2* gene model was improved using RNA-seq data (Kim *et al.* 2013).

The EquCab2.0 assembly is highly fragmented over the PAR and therefore, identifying the Del-1 and Del-2 breakpoints from short read alignments proved futile. We attempted *de novo* assemblies using short reads from SA cases, which resulted in very fragmented assemblies. As an alternate strategy, we applied single-molecule real-time (SMRT) sequencing technology (PacBio) to sequence large-insert BAC clones generated from the equine CHORI-241 BAC library (http://bacpac.chori.org). We selected nine clones whose end sequence alignments formed a tiling path along the PAR on both sides of the *SHOX* coding sequence (Table 1, Table S3, and Figure S1). After quality control, error-correction, and assembly we merged contigs based on overlapping similarities in relation to EquCab2.0. We based downstream analyses on the sequence assembly of three BAC-derived consensus contigs generated from five sequenced BAC clones (Table S4 and Figure 4A).

**Table 1:** BAC clone assemblies generated from SMRT sequencing data (see Table S3 for detailed information)

BAC Clones	Assembly Size (bp)	#Contigs	GC*^a^*%	Assembly Fraction*^b^* %
194E12*^c^*	155,628	1	59.7	72
288L23*^c^*	186,195	7	58.3	NA
50P17*^c^*	147,467	1	58.6	70
52P20	66,939	2	54.4	34
159K1*^c^*	47,668	4	53.5	NA
442L16	58,892	1	55.9	31
291B18	107,104	3	54.7	60
712C2*^c^*	140,175	1	55.8	NA
419P11*^c^*	73,186	1	57.9	37

a G/C nucleotide content of assembly.

b Fraction of BAC assembled based on anticipated size from BAC end sequence alignment. NA = not applicable because the anticipated size of the BAC is not known.

c These BACs were included in the previously published PAR BAC contig map (Raudsepp and Chowdhary 2008).

We generated an alternative genome assembly by merging these BAC-derived consensus sequences together with the EquCab2.0, excluding parts of EquCab2.0 represented also in the BAC-derived consensus sequences.

### Detection of deletion breakpoints

We next aligned the short reads from all cases and the control pool to this alternative genome assembly, determined copy numbers (Figure 4B) and called SNPs using stringent filtering. The Del-1 depth pattern, *i.e.*, complete lack of aligned reads for cases 1, 5, and 6, an approximate 50% reduction in depth for cases 2, 3, and 4, and normal depth in the control pool, was observed for ∼97 kb on BAC-C1 (BAC-C1 ≈133,000−230,000 bp). A presumed breakpoint (BP1) (Figure 4B) flanked by a stretch of a TGGA repeat was observed in this contig. In the other two BAC-derived sequences (BAC-C2 and BAC-C3) we identified regions showing the Del-2 pattern (*i.e.*, all the cases lacked depth while the control pool had normal depth). Interestingly, within the Del-2 region of BAC-C3 we identified a coding sequence showing partial similarity to human *CRLF2* (Figure 4B). This gene is located downstream of *SHOX* in human and horse (Figure 3) (Raudsepp and Chowdhary 2008; Raudsepp *et al.* 2012). We improved the annotation of this gene by using previously published RNA-seq data from Thoroughbred horses (Kim *et al.* 2013).

Based on these observations we estimated Del-2 to encompass 60−80 kb of the genome, with the size uncertainty being a result of an evident mis-assembly in BAC-C2. We observed an enrichment of soft-clipped reads in the control pool immediately adjacent to BP2 (BAC-C2: 28,000−30,000 bp), which we were unable to amplify over by PCR. In addition, there was not a single read pair bridging over this problematic region in the control pool.

As was the case for BP1 of Del-1 on BAC-C1, the sequence flanking BP3 on BAC-C3 featured a stretch of TGGA repeats longer than 1 kb. Because of these repeats, we could not characterize the breakpoints using mapping data of the paired-end Illumina reads from the sequenced SA cases. Using DNA from SA cases we attempted PCR-based chromosome walking to extend this region into the downstream sequence of the deletion but these efforts proved futile, possibly due to complexity of this region, such as high repeat and G/C nucleotide content.

### Diagnostic tests for Del-1 and Del-2

**Figure 5:**
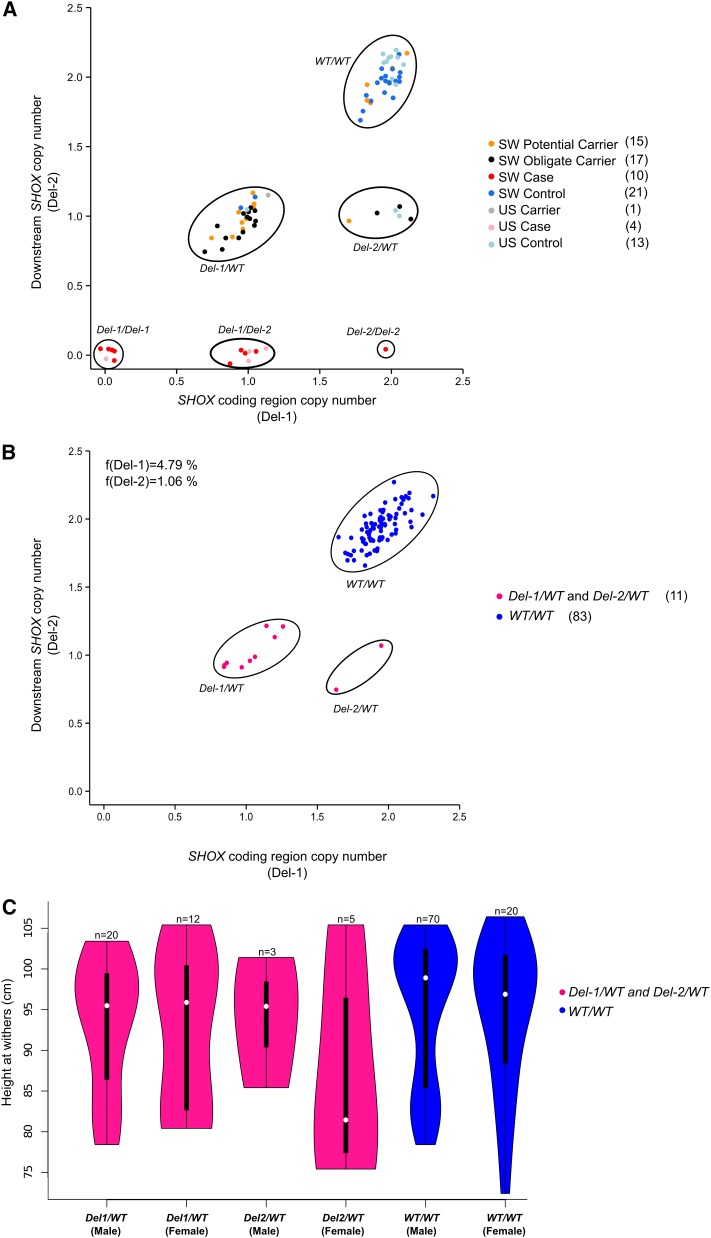
Diagnostic test for Del-1 and Del-2. (A) TaqMan genomic copy number assay results from analysis of cases, carriers, and controls from Sweden (SW) and the USA (US). Potential carriers are parents to unverified cases or close relatives to verified carriers and obligate carriers are known to have produced confirmed atavistic offspring. Numbers of individuals genotyped in each group are presented in parentheses to the right. (B) TaqMan genomic copy number assay results from analysis of 94 individuals randomly selected from the Swedish Shetland pony population together with observed allele frequencies. (C) Height at withers distribution subdivided by sex and genotype. For this analysis we included all genotyped individuals with height and sex data available (except cases) and seven carriers that had not been genotyped but whose genotype could be inferred by pedigree information.

In order to validate findings from the sequencing efforts and to estimate Del-1 and Del-2 allele frequencies we designed TaqMan copy number assays for genotyping by droplet digital PCR (ddPCR). We genotyped two sets of samples: (i) cases, obligate carriers, potential carriers being close relatives to known carriers or having had unconfirmed affected foals, and control individuals without history of having sired any affected foals (in total 63 Swedish Shetland ponies and 18 American miniature horses); and (ii) a random set of Swedish Shetland ponies. Table S5 and Figure 5A show the results of genotyping. All SA cases but no controls nor carriers were homozygous *Del-1*, homozygous *Del-2*, or hemizygous *Del-1/Del-2*. Most control individuals formed a separate cluster showing two copies for each targeted locus while obligate carriers were genotyped as either *Del-1/WT* or *Del-2/WT*. Ten individuals in the potential carrier group clustered with obligate carriers as *Del-1/WT* or *Del-2/WT* while five of them were *WT/WT*. In the random set of 94 Swedish Shetland ponies, 11.7% of horses were identified as *Del-1* or *Del-2* carriers. In this randomly selected set of samples we observed carriers (*n* = 11) and *WT* allele homozygotes (*n* = 83) but no *Del-1* or *Del-2* homozygotes nor compound *Del-1*/*Del-2* heterozygotes (Figure 5B and Table S5). Based on genotypes observed in the random set of samples we estimated the allele frequencies of *Del-1* and *Del-2* to be 4.79% and 1.06%, respectively, in the Swedish Shetland pony population at the time of sampling, between 1968 and 2000 (70% of the individuals were born in the 1990s).

In the current set of samples (*n*_(total)_ = 130), we examined the association of height at withers with deletion genotypes. No significant difference in height could be revealed between carriers [*Del-1/WT* (*n* = 32) and *Del-2/WT* (*n* = 8)] and *WT/WT* (*n* = 90) when considering sex as covariate (ANOVA *P* = 0.25; Figure 5C). Furthermore, no significant association with height was found for the three genotypes (*Del-1/WT*, *Del-2/WT*, and *WT/WT*) and sex (*P* = 0.31).

## Discussion

GWAS based on the EquineSNP50 BeadChip did not reveal any association and we conclude that this was because only anchored chromosomes were used for selection of SNPs in the early BeadChip versions. Here we performed whole genome resequencing of six cases and a DNA pool of control individuals and revealed two causal variants; two large deletions (∼160 and ∼80 kb in size) of sequences only partially represented in the EquCab2.0 assembly as unanchored scaffolds, occurring in conjunction with and overlapping the *SHOX* gene, respectively. Three of the sequenced cases were homozygous for *Del-1*, which spans the entire *SHOX* gene, while the other three were heterozygous *Del-1/Del-2*, but due to partial overlap of the deletions all cases shared 60−80 kb of deleted sequence downstream of *SHOX* including the deletion of another gene (*CRLF2*). In the BAC assemblies we did not observe any sequence unique to Del-2, which appears to completely correspond to the distal part of Del-1. By sequencing and assembling BAC clones we could order contigs and annotate the *CRLF2* gene more accurately than in EquCab2.0, which was quite fragmented in this region.

*SHOX* expression is crucial during development, as SHOX is involved in cell cycle regulation and acts as a transcriptional activator in osteogenic cells (Rao *et al.* 2001). Regulation of *SHOX* expression is complex at the transcriptional and translational levels (Blaschke and Rappold 2006), impairment of regulatory elements can affect its expression, and missense and nonsense mutations have also been reported to alter the structure and consequently the activity of the protein (Ross *et al.* 2001; Jorge *et al.* 2007; Chen *et al.* 2009; Benito-Sanz *et al.* 2012). Deficiency and haploinsufficiency of *SHOX* have been associated with skeletal defects involving short stature and limb deformities in humans (Jorge *et al.* 2007; Chen *et al.* 2009; Raudsepp *et al.* 2012). Benito-Sanz *et al.* (2012) identified a 47-kb deletion downstream of *SHOX* as the disease-causing allele in Léri-Weill dyschondrosteosis (LWD) and idiopathic short stature (ISS) patients. Chen *et al.* (2009) also reported that microdeletions located 250 kb downstream of this gene can lead to short stature.

In addition to the homozygous/hemizygous deletion of the coding sequence of *SHOX*, all the SA cases had homozygous deletions over the predicted coding sequence of *CRLF2*. The CRLF2 protein is a type 1 cytokine receptor involved in hematopoietic cell development and in the JAK-STAT pathway, active in bone metabolism and development (Al-Shami *et al.* 2004; Li 2013; Hanada *et al.* 2014). Poggi *et al.* (2015) reported large deletions in PAR spanning *SHOX* and *CRLF2* in some of the LWD patients, but the common feature among LWD cases is deletions spanning *SHOX*. Thus, although we cannot exclude that the deletion of *CRLF2* is involved in the phenotypic expression of SA, we argue that the similarity to human phenotypes caused by *SHOX* deficiency support the interpretation that SA is manifested by a reduction of *SHOX* expression. The deletion of *CRLF2* may affect other hitherto unexplored phenotypes in affected horses and possibly also in carriers.

*SHOX* and *CRLF2* are both located in the PAR, which like autosomes experiences recombination in both sexes, and is not subjected to X-inactivation in females (Brown and Greally 2003). This region has the highest recombination rate in the human genome and a high incidence of structural changes (Blaschke and Rappold 2006). The high G/C nucleotide content and repeat content of the PAR can explain the difficulties in sequencing and assembling this part of the genome, especially using short read sequencing data. Even in human, because of these complexities, ∼600 kb of PAR has still not been assembled (Blaschke and Rappold 2006). However, these complex regions can be better resolved by utilizing long-read sequencing data (Huddleston *et al.* 2014). By using SMRT sequencing we were able to considerably improve the assembly of the *SHOX* locus in horse. Despite this improvement, it still proved challenging to resolve and precisely identify the deletion breakpoints. Our results show that the breakpoints are enriched by long stretches of microsatellites (TGGA)n and this type of repeat can cause chromosomal rearrangements (Bena *et al.* 2010). Expanded pairs of homologous repeats, such as (TGG)n, form strong secondary structures inhibiting DNA synthesis as length of the repeat increases (Usdin 1998). For instance, a 1.11-Mb deletion on human 14q32 has been shown to be mediated by such a repeat (Bena *et al.* 2010).

In addition, these repeats are unstable, leading to replication slippage or increased chance of recombination by which new structural changes can be introduced (Fry and Usdin 2006). Repeat instability is a complex feature of the genome and is expected to be influenced by many factors and pathways. Our efforts to bridge across the breakpoints failed, likely due to complex structure of this region inhibiting DNA polymerase activity. The best approach at hand to resolve the breakpoints may be to conduct long-read single-molecule sequencing of the entire genome or isolated sex chromosomes from an affected individual. In the absence of such a resource we designed TaqMan copy number assays to genotype *Del-1* and *Del-2* in cases, carriers, and controls as well as a population sample. Our genotyping showed independent clustering of affected individuals (*Del-1/Del-1*, *Del-1/Del-2*, and *Del-2/Del-2*), carriers (*Del-1/WT* and *Del-2/WT*), and control individuals (*WT/WT*). Among the genotyped individuals we only observed one *Del-2/Del-2* individual (Figure 5A), which was not surprising due to the low allele frequency of *Del-2* observed in the random set.

We did not observe any obvious phenotypic difference between *Del-1/Del-1* and *Del-1/Del-2* individuals. More detailed bone and cartilage phenotyping may make it possible to characterize whether there is a difference between *Del-1/Del-1* and *Del-1/Del-2* individuals. Since all individuals carrying two deletion alleles manifest an identical or at least very similar disorder but only Del-1 removes the *SHOX* coding sequence, we expect the *Del-2* deleted region to contain regulatory elements governing *SHOX* expression during development. However, alignment of the Del-2 region on the human genome did not overlap any annotated regulatory elements. Such regulatory elements may however still be unannotated in humans, may be unique to horse, or may be missing from the SMRT assembly since some BACs were assembled into contigs covering less than 50% of the estimated size of the BAC clones. Thus, to characterize the presence of such elements more precisely, one would have to improve the assembly further and conduct functional genomics screens.

Historically in Shetland pony breeding, small size combined with strength and hardiness have been important traits under selection. Using a small sample set of carriers we did not observe any significant association of this locus with height, but being restricted to a small sample set of carriers we cannot exclude the possibility that the lack of association was due to poor power to detect a true effect on height. It is also possible that certain phenotypes associated with SA cases, for example differences in phenotypic expression between *Del-1* homozygotes and *Del-1/Del-2* hemizygotes, or deletion carriers might have been overlooked. Our findings can now be directly applied in breeding programs to avoid foals born with skeletal atavism.

## Supplementary Material

Supplemental Material
