# Supplementary material for: Large Deletions at the SHOX Locus in the Pseudoautosomal Region Are Associated with Skeletal Atavism in Shetland Ponies
Source: G3 (Bethesda). 2016 May 19;6(7):2213–23. doi: 10.1534/g3.116.029645 (PMC4938674; doi:10.1534/g3.116.029645)
Supplement: Supplemental Material [file supp_6_7_2213__index.html]

Large Deletions at the SHOX Locus in the Pseudoautosomal Region Are Associated with Skeletal Atavism in Shetland Ponies — Supplemental Material 

# Large Deletions at the SHOX Locus in the Pseudoautosomal Region Are Associated with Skeletal Atavism in Shetland Ponies

## Supplemental Material for Rafati, *et al*, 2016

**Files in this Data Supplement:**

- Table S1 - Sex, sequencing depth and proportions of reads mapping to EquCab2.0 for sequenced cases and control pool. (PDF, 45 KB)
- Table S2 - Assembly statistics of regions with signature of deletion (The coordinates are based on UCSC genome browser concatenation of unassigned scaffolds). (PDF, 59 KB)
- Table S3 - Detailed information of BAC clone assemblies generated from SMRT sequencing data. (PDF, 59 KB)
- Table S4 - BAC-derived consensus contigs statistics. (PDF, 46 KB)
- Table S5 - Trait classes by genotype. (PDF, 65 KB)
- Table S6 - Sequences of primers and probes used in digital droplet PCR. (.pdf, 53.2 KB)
- Figure S1 - BAC clone coordinates on chrUn (see Raudsepp et al., 2012). (.pdf, 100 KB)
