# Supplementary material for: Large Deletions at the SHOX Locus in the Pseudoautosomal Region Are Associated with Skeletal Atavism in Shetland Ponies
Source: G3 (Bethesda). 2016 May 19;6(7):2213–23. doi: 10.1534/g3.116.029645 (PMC4938674; doi:10.1534/g3.116.029645)
Supplement: Supplemental Material [file supp_g3.116.029645_Table_S1.pdf]

Table S1. Sex, sequencing depth and proportions of reads mapping to EquCab2.0 for sequenced cases and control pool.

Sample	Sex	Coverage	Mapped reads%
Case 1	F	~ 7x	95
Case 2	F	~ 7x	95
Case 3	F	~ 7x	95
Case 4	M	~ 7x	97
Case 5	F	~ 7x	94
Case 6	M	~ 7x	95
Pool of controls		~ 56x	98
