# Supplementary material for: Large Deletions at the SHOX Locus in the Pseudoautosomal Region Are Associated with Skeletal Atavism in Shetland Ponies
Source: G3 (Bethesda). 2016 May 19;6(7):2213–23. doi: 10.1534/g3.116.029645 (PMC4938674; doi:10.1534/g3.116.029645)
Supplement: Supplemental Material [file supp_g3.116.029645_Table_S2.pdf]

Table S2. Assembly statistics of regions with signature of deletion (The coordinates are based on UCSC genome browser concatenation of unassigned scaffolds).

Chromosome	Start	End	scaffold	Size[a]	#contigs	Min[b] (bp)	Max[c] (bp)	GC %	Locus[d]
1[e]	23,547,000	23,549,000	scaffold_5	2,000	1	78,820	78,820	37.23	Del-1
Un	26,645,953	26,647,166	chrUn0036	1,214	1	1,399	27,363	50.74	Del-1
Un	26,650,752	26,745,752	chrUn0036	59,908	12	1,399	27,363	58.10	Del-1
Un	26,747,752	26,779,752	chrUn0036	15,671	6	783	7,114	56.67	Del-2
Un	31,010,752	31,011,752	chrUn0050	1,000	1	30,216	30,216	52.36	Del-1
Un	49,746,752	49,806,752	chrUn0189	51,310	8	1,984	19,319	56.11	Del-2
Un	50,401,752	50,404,752	chrUn0198	2,000	1	30,666	30,666	52.48	Del-1
Un	53,315,752	53,319,752	chrUn0250	4,000	1	27,756	27,756	52.60	Del-1
Un	62,955,752	62,957,752	chrUn0568	1,758	1	8,549	8,549	51.62	Del-1
Un	65,524,752	65,525,752	chrUn0717	1,000	1	8,048	8,048	51.22	Del-1
Un	67,675,752	67,684,752	chrUn0874	6,000	3	1,555	3,101	49.73	Del-1
Un	69,440,752	69,454,752	chrUn1005	6,860	4	1,396	3,015	53.90	Del-2
Un	93,181,752	93,184,752	chrUn4326	2,731	1	4,165	4,165	50.62	Del-1
Un	103,088,752	103,094,752	chrUn6292	3,503	2	1,516	1,987	54.18	Del-2

[a] Size of deleted sequence.
[b] Minimum size of contig in given coordinate.
[c] Maximum size of contig in given coordinate.
[d] Del-1: Regions where three of the cases (Case1, Case5, Case6) show deletion. Del-2: Regions where all cases show deletion.
[e] 90% of this region is repetitive elements (SINE/LINE).
