# Supplementary material for: Large Deletions at the SHOX Locus in the Pseudoautosomal Region Are Associated with Skeletal Atavism in Shetland Ponies
Source: G3 (Bethesda). 2016 May 19;6(7):2213–23. doi: 10.1534/g3.116.029645 (PMC4938674; doi:10.1534/g3.116.029645)
Supplement: Supplemental Material [file supp_g3.116.029645_Table_S3.pdf]

Table S3. Detailed information of BAC clone assemblies generated from SMRT sequencing data.

BAC Clones	Assembly Size (bp)	#Scaffolds	Min (bp)	Max (bp)	GC[a]%	Lab.[b]	Assembly Fraction[c] (%)
194E12 [d]	155,628	1	155,628	155,628	59.7	WSM	72
288L23 [d]	186,195	7	886	57,130	58.3	SciLife	NA
50P17 [d]	147,467	1	147,467	147,467	58.6	WSM	70
52P20	66,939	2	852	66,087	54.4	SciLife	34
159K1 [d]	47,668	4	1,660	27,250	53.5	SciLife	NA
442L16	58,892	1	58,892	58,892	55.9	WSM	31
291B18 (1)	107,104	3	23,331	45,533	54.7	WSM	60
291B18 (2)	67,454	2	899	66,555	53.5	SciLife	38
712C2 [d]	140,175	1	140,175	140,175	55.8	WSM	NA
419P11 [d]	73,186	1	73,186	73,186	57.9	WSM	37

[a] GC content of assembly.
[b] Labs that sequenced BAC clones. WSM: Washington School of Medicine, Washington, United States of America. SciLife: Science for Life laboratory, Uppsala, Sweden.
[c] Fraction of BAC assembled based on anticipated size from BAC end sequence alignment. NA=not applicable because the anticipated size of the BAC is not known.
[d] These BACs were included in the previously published PAR BAC contig map (see Raudsepp et al., 2008).
