# Supplementary material for: Large Deletions at the SHOX Locus in the Pseudoautosomal Region Are Associated with Skeletal Atavism in Shetland Ponies
Source: G3 (Bethesda). 2016 May 19;6(7):2213–23. doi: 10.1534/g3.116.029645 (PMC4938674; doi:10.1534/g3.116.029645)
Supplement: Supplemental Material [file supp_g3.116.029645_Table_S4.pdf]

Table S4. BAC-derived consensus contigs statistics.

Contig	BAC clone	Size (bp)	GC%
**BAC-C1**	194E12 (1[a])<br>50P17 (1)<br>291B18 (3)	240,449	58.53
**BAC-C2**	52P20	44,256	56.64
**BAC-C3**	712C2	140,175	55.80

[a] Number in parentheses shows numbers of contigs used to generate BAC-derived contigs
