# Supplementary material for: Large Deletions at the SHOX Locus in the Pseudoautosomal Region Are Associated with Skeletal Atavism in Shetland Ponies
Source: G3 (Bethesda). 2016 May 19;6(7):2213–23. doi: 10.1534/g3.116.029645 (PMC4938674; doi:10.1534/g3.116.029645)
Supplement: Supplemental Material [file supp_g3.116.029645_Table_S5.pdf]

Table S5. Trait classes by genotype. For the Swedish sample set: Horses with unknown phenotype were selected randomly from samples available at Animal Breeding and Genetics, SLU. Control horses were selected if they met the criteria of siring more than 50 non-atavistic offspring. Obligate carriers were individuals with history of having atavistic offspring. Figures in parentheses denotes obligate carriers with the genotype inferred (including both parents and grandparents of cases). Potential carriers were chosen either because a) they were close relatives to known carriers or b) suspected to be carriers because they were parents to unconfirmed cases. Phenotypes of affected horses were confirmed by X-ray, veterinarian examination and/or photography. For the US sample set, cases were identified by horse owners and samples submitted in connection with investigations of another form of dwarfism. Photographs were used to identify atavistic samples to confirm phenotypic characteristics.

		WT/WT	***Del-1/WT***	***Del-2/WT***	***Del-1/Del-1***	***Del-1/Del-2***	***Del-2/Del-2***	**Total**
**Swedish**	**Affected horses**	-	-	-	5	4	1	10
	**Obligate carriers**	-	14 (+5)	3 (+2)	-	-	-	17 (+7)
	**Potential carriers**	5	9	1	-	-	-	15
	**Control horses**	19	2	-	-	-	-	21
	**Randomly selected horses**	83	9	2	-	-	-	94
**American**	**Affected horses**	-	-	-	1	3	-	4
	**Obligate carriers**	-	1	-	-	-	-	1
	**Potential carriers**	-	-	-	-	-	-	0
	**Control horses**	10	1	2	-	-	-	13
	**Randomly selected horses**	-	-	-	-	-	-	0
