# Supplementary material for: Large Deletions at the SHOX Locus in the Pseudoautosomal Region Are Associated with Skeletal Atavism in Shetland Ponies
Source: G3 (Bethesda). 2016 May 19;6(7):2213–23. doi: 10.1534/g3.116.029645 (PMC4938674; doi:10.1534/g3.116.029645)
Supplement: Supplemental Material [file supp_g3.116.029645_TableS6.pdf]

Table S6. Sequences of primers and probes used in digital droplet PCR.

Name	Type	Target	Sequence
MST3-F	Forward	Myostatin	TGCAACACTGTCTTCACATCAATG
MST3-R	Reverse	Myostatin	TCCGATCTCTGAAACTTGACATGA
Del-1-1-F	Forward	Del-1	CCAGAGCAACGGCAAGKA
Del-1-1-R	Reverse	Del-1	AAATGCAAGGTCGCACCAA
Del-1-3-F	Forward	Del-1	CTCAGCCGCCGACAACA
Del-1-3-R	Reverse	Del-1	TGTTACTGTGCGTTTGAGACGTT
Del-1-4-F	Forward	Del-1	GAAAGAGCCGGGAGGCA
Del-1-4-R	Reverse	Del-1	CCCTGTGTTCTGAGTGCGACT
Del-2-1-F	Forward	Del-2	TTATACAAGTCCACGCCTTTGGT
Del-2-1-R	Reverse	Del-2	CGCGACCGGTGGTGTT
Del-2-3-F	Forward	Del-2	GGGTTCCTCGATTGACTTTATGA
Del-2-3-R	Reverse	Del-2	CGTTTGAGAAGCGGCGATT
Del-2-4-F	Forward	Del-2	TGCAGAGTCGACACCAAGTCA
Del-2-4-R	Reverse	Del-2	TGCCGGGTGCAAACGT
Old-Del-1-F	Forward	Del-1	TCCCCGRGTGTGGAAAGTTA
Old-Del-1-R	Reverse	Del-1	CCACAAAGCACATCCGTTTA
Old-Del-2-F	Forward	Del-2	CCMGCTTTTGTCCCTTAAAC
Old-Del-2-R	Reverse	Del-2	TCCAGGCGATTTCCAACTAA
RNAseP-F	Forward	RNAseP	GTTCCAAGCTCCGGCTAAG
RNAseP-R	Reverse	RNAseP	GGAGGTGGGTTCCCAGAG
MST3P [a]	Probe	Myostatin	TCTGCCAAATACCAGCGCCTGGG
Del-1-1-P [b]	Probe	Del-1	ATGACTAACTGTTGACTTCG
Del-1-3-P [b]	Probe	Del-1	CTTCCTGCGACGTTTA
Del-1-4-P [b]	Probe	Del-1	CGTCCACTCTCAGTCGT
Del-2-1-P [b]	Probe	Del-2	CCCGGAAGCTCACA
Del-2-3-P [b]	Probe	Del-2	ACAGTGAAGTGTGACTATG
Del-2-4-P [b]	Probe	Del-2	CTGAGCCGCGCGTC
RNAseP-P [a]	Probe	RNAseP	TCTGCCCTCGCGCGGAGC
Old-Del-1-P [b]	Probe	Del-1	ACGGGAAGGAGGGGGCCC
Old-Del-2-P [b]	Probe	Del-2	CCAGCTCTGGGCTCGGCT CC

a 5’ labeled with VIC and 3’ labeled with TAMRA.
b 5’ labeled with 6-FAM and 3’ labeled with the minor groove binder (MGB) non-fluorescent quencher.
