# Supplementary figures and images for: Large Deletions at the SHOX Locus in the Pseudoautosomal Region Are Associated with Skeletal Atavism in Shetland Ponies
Source: G3 (Bethesda). 2016 May 19;6(7):2213–23. doi: 10.1534/g3.116.029645 (PMC4938674; doi:10.1534/g3.116.029645)

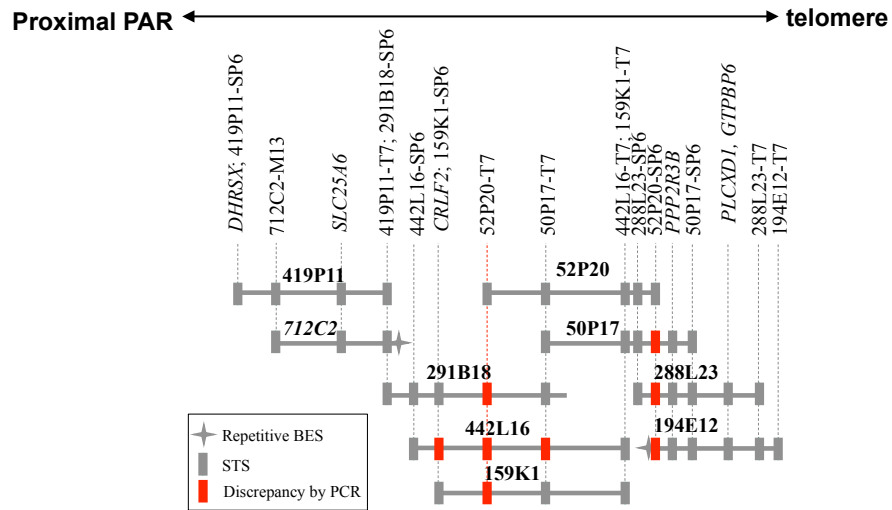

**Figure S1.** BAC clone coordinates on chrUn (see Raudsepp et al., 2012)

Supplement: Supplemental Material [file supp_g3.116.029645_FigureS1.pdf]
